# Double valve replacement for acute spontaneous left chordal rupture secondary to chronic aortic incompetence

**DOI:** 10.1186/1749-8090-1-33

**Published:** 2006-10-06

**Authors:** Sandeep Agarwala, Sanjay Kumar, John Berridge, Jim McLenachan, David J O'Regan

**Affiliations:** 1 Department of Cardiothoracic surgery, Yorkshire Heart Centre, Leeds General Infirmary, Leeds, UK; 2 Department of Anesthesiology, Yorkshire Heart Centre, Leeds General Infirmary, Leeds, UK; 3 Department of Cardiology, Yorkshire Heart Centre, Leeds General Infirmary, Leeds, UK; 4 Consultant Cardiothoracic Surgeon, Yorkshire Heart Centre, D Floor, Jubilee Building, Leeds General Infirmary, Great George Street, Leeds, LS1 3EX, UK

## Abstract

A 54 years old male with undiagnosed chronic calcific degenerative aortic valve incompetence presented with acute left anterior chordae tendinae rupture resulting in severe left heart failure and cardiogenic shock. He was successfully treated with emergency double valve replacement using mechanical valves. The pathogenesis of acute rupture of the anterior chordae tendinae, without any evidence of infective endocarditis or ischemic heart disease seems to have been attrition of the subvalvular mitral apparatus by the chronic regurgitant jet of aortic incompetence with chronic volume overload. We review the literature with specific focus on the occurrence of this unusual event.

## Background

Acute spontaneous rupture of the anterior chordae tendinae of a normal mitral valve leading to acute mitral incompetence is uncommon. This report is the first documentation of tricuspid calcific degeneration of the aortic valve with chronic incompetence leading to acute chordal rupture of the anterior mitral leaflet (AML).

## Case summary

A 54 year old male presented with sudden onset chest pain, dyspnoea and palpitations. There was no previous history of cardiac illness; in particular there was no history of rheumatic fever. He was a non smoker with no risk factors for ischemic heart disease. There was no past history of chronic renal failure, primary or secondary hyperthyroidism. His initial clinical examination revealed him to be in atrial fibrillation with a fast ventricular rate. His blood pressure was 106/72 mm Hg. He had an elevated jugular venous pressure and a harsh systolic murmur over the precordium. The respiratory rate was 40/min and there were fine crepitations throughout the lung fields. His oxygen saturation was 96% on 10 litres oxygen. The chest x-ray confirmed pulmonary oedema. His Electrocardiogram showed voltage evidence of left ventricular hypertrophy with volume overload, but no evidence of acute, or old myocardial infarction. His basic blood investigations, creatinine kinase (CK-MB) and troponin I levels were normal.

Despite optimisation of medical therapy, his condition deteriorated over the next four hours. He developed cardiogenic shock with a BP of 80/50 mm Hg and oliguria; he required CPAP to maintain his oxygen saturation. Subsequently he was sedated, intubated and ventilated. The treatment with intravenous Amiodarone restored sinus rhythm.

**Figure 1:**
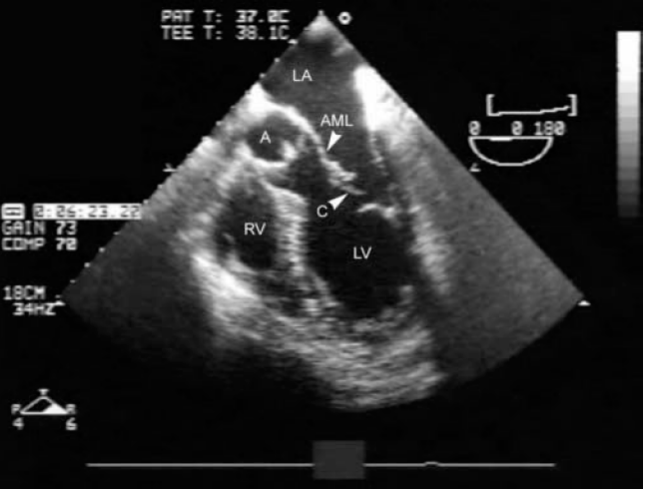
Transesophageal Echocardiogram showing ruptured chordae (C) of anterior mitral leaflet (AML). Left ventricle (LV) and Left atrium (LA), Right ventricle (RV) and aortic valve (A) are shown.

**Figure 2:**
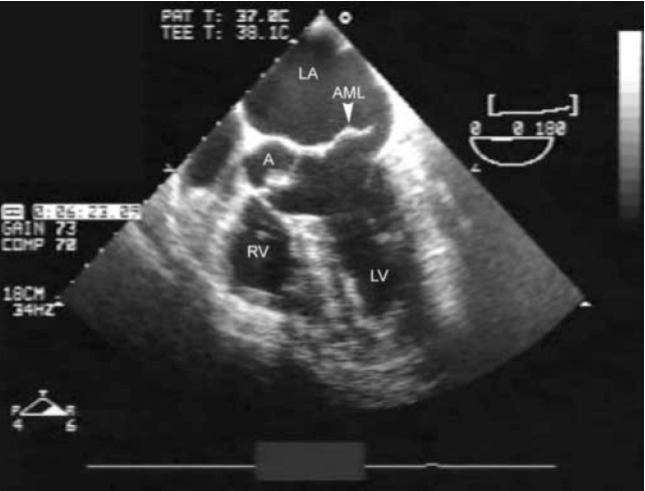
Transesophageal Echocardiogram shows ruptured chordae (C) of anterior mitral leaflet (AML) prolapsing into LA.

A pulmonary artery floatation catheter was inserted at this stage and confirmed cardiogenic pulmonary oedema with Cardiac Index of 1.2, pulmonary artery occlusion pressure of 30 mmHg with a prominent v wave. A transesophageal echocardiogram showed severe mitral incompetence due to chordal rupture with a flail anterior mitral leaflet (Figure 1) prolapsing into the left atrium (Figure 2). The aortic valve leaflets were noted to be thickened with mild-moderate aortic regurgitation. The left ventricle was dilated with end systolic dimension in the transgastric mid short axis view being 4.7 cms.

It was decided to undertake urgent mitral valve replacement to manage his acute pulmonary oedema and cardiogenic shock. The cardiopulmonary bypass (CPB) was established with aorto-bicaval cannulation and patient was cooled to 28 degree Celsius. An apical left ventricular vent was placed. Severe aortic incompetence was noted during cardioplegia delivery. The aorta was opened and subsequent myocardial protection was achieved by cold intermittent antegrade cardioplegia through the coronary ostia.

The aortic valve was found to be tricuspid, but thickened with calcific degeneration and was grossly incompetent. The aortic root appeared ectatic with some dilatation of the root and the sinuses of valsalva. The coronary ostia were normally placed. An atriotomy was made in a mildly enlarged left atrium behind the interatrial groove. On inspection of the mitral valve, all rough zone chordae of the AML including strut chordal attachment to papillary muscle were ruptured and the AML was completely flail with free prolapse into left atrium. The posterior mitral leaflet (PML) appeared normal and its subvalvar apparatus was preserved. The aortic and mitral valves were replaced using a 25 mm Carbomedics Tophat and 31 mm Carbomedics mechanical valve prosthesis respectively. The patient was weaned off CPB easily in normal sinus rhythm. The cross clamp time was 116 minutes and the CPB time was 201 minutes.

**Figure 3:**
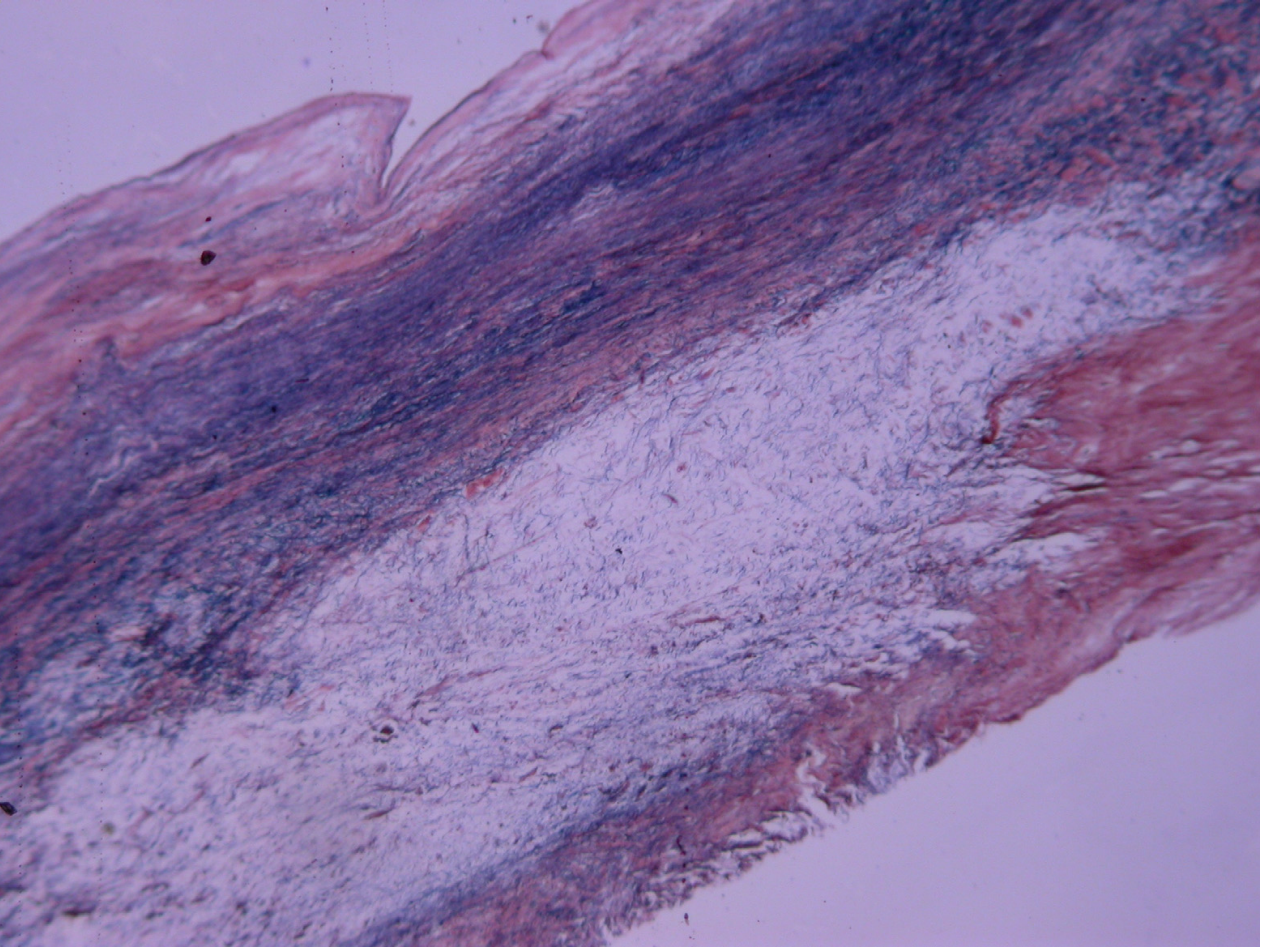
Elastin Van Gieson staining of the leaflet and chordae showing central myxoid degeneration with chronic superficial fibrosis.

Postoperatively, ventilation was required for 48 hours and extubation was uneventful. There was a transient rise in creatinine to 219 μmol/l (ref range 70–100) without significant oliguria and he did not require renal replacement therapy. Creatinine level normalised to 107 μmol/l by day four and he was discharged on the seventh postoperative day. Histopathological examination of the excised valves and chordae showed central myxoid degeneration with chronic superficial fibrosis, [Figure 3] without the underlying cause being apparent.

## Discussion

Acute mitral incompetence is due to failure of the valve mechanism at annulus, leaflet or subvalvar level. Chordal rupture is the least commonly encountered pathology [1]. It may be idiopathic or secondary to myxomatous degeneration, underlying papillary muscle fibrosis or dysfunction, infective endocarditis or connective tissue disorders. Incidental chordal rupture is seen in 12% of the patients undergoing mitral surgery but less than 4% of these present as acute mitral regurgitation in pulmonary oedema and cardiogenic shock [2]. This usually involves single chordae of the PML and leads to mitral prolapse with insidious onset regurgitation which progresses with time. Anterior chordal involvement is uncommon [2].

Acute chordal rupture has been described in association with acquired aortic stenosis [3]. In this condition, mitral annular calcification, ventricular anatomic and haemodynamic alterations may contribute to the rupture. Congenital aortic valve disease leading to chordal rupture has also been described. [4] Chordal rupture is known to occur in patients with hypertrophic obstructive cardiomyopathy and is due to the abnormal systolic anterior motion of the anterior mitral leaflet [5]. A tricuspid aortic valve with calcific degeneration and severe aortic incompetence leading to acute chordal rupture of the AML has not previously been reported.

The presence of left ventricular hypertrophy on the electrocardiogram and the cardiomegaly with left ventricular configuration on radiological examination indicates the chronic nature of the aortic incompetence. In the present case, we suggest that the chronic jet of aortic valvular incompetence hitting the AML subvalvar apparatus along with left ventricular dilatation due to volume overload led to the elongation and thinning of the chordae, which ruptured at the attachment site leading to torrential mitral incompetence. This was evident as the ruptured chordae showed chronic fibrotic changes on histology. This event led to marked elevation of left ventricular filling pressures, left atrial mean pressures and a reduction of the cardiac output. The aortic regurgitation could have deteriorated and this resulted in acute left ventricular failure.

Valvuloplasty may be a procedure of choice for selected patients with ruptured chordae to the AML in patients with isolated mitral incompetence [6]. Mitral valve repair as a technically viable option in an unstable patient with complete rupture of chordae of AML can be debated. Valve replacement is the most widely accepted solution when there is severe rupture of the chordae, as in the present case, along with concomitant aortic valve pathology. The mortality of these patients with cardiogenic shock is extremely high and, therefore, early diagnosis and immediate surgery is crucial [4]. Emergency valve replacement provides excellent results [4]. The choice of mechanical prostheses in the present case is consistent with his young age.
